# Antioxidant Compound Adsorption in Polyvinylpolypyrrolidone from Chilean *Carménère, Cabernet Sauvignon,* and *Merlot* Grape Pomaces as Potential By-Products

**DOI:** 10.3390/antiox11102017

**Published:** 2022-10-12

**Authors:** Nelson Díaz, Pedro M. Aqueveque, Alejandro Vallejos-Almirall, Rudi Radrigán, María C. Zúñiga-López, Christian Folch-Cano

**Affiliations:** 1 Departamento de Agroindustrias, Facultad de Ingeniería Agrícola, Universidad de Concepción, Av. Vicente Méndez 595, Chillán 3812120, Chile; 2 Grupo Interdisciplinario de Biotecnología Marina (GIBMAR), Centro de Biotecnología, Universidad de Concepción, Edmundo Larenas 64, Concepción 4070386, Chile; 3 Centro de Desarrollo Tecnológico Agroindustrial (CDTA), Facultad de Ingeniería Agrícola, Universidad de Concepción, Av. Vicente Méndez 595, Chillán 3812120, Chile; 4 Departamento de Química Inorgánica y Analítica, Facultad de Ciencias Químicas y Farmacéuticas, Universidad de Chile, Sergio Livingstone 1007, Santiago 8380492, Chile

**Keywords:** grape pomace, phenolic compounds, polyvinylpolypyrrolidone, adsorption, anti-bacteria

## Abstract

Grape pomace (GP) is a by-product resulting from the winemaking process and its potential use as a source of bioactive compounds is well known. The GP bioactive compounds can be retained in the well-known polyvinylpolypyrrolidone (PVPP), industrially used in the clarification and stabilization of wine and other drinks. Thus, the polyphenolic compounds (PC) from the Chilean *Carménère*, *Cabernet Sauvignon*, and *Merlot* GP were extracted, and their compositions and antioxidant capacities (ORAC-FL) were determined. In addition, the retention capacity of the PC on PVPP (PC-PVPP) was evaluated. The bioactivities of GP extracts and PC-PVPP were estimated by the agar plate inhibition assay against pathogenic microorganisms. Results showed a high amount of TPC and antioxidant capacity in the three ethanolic GPs extracts. Anthocyanins, flavan-3-ol, and flavonols were the most abundant compounds in the GP extract, with retentions between 70 and 99% on PVPP. The GP extracts showed inhibition activity against *B. cereus* and *P. syringae* pv. *actinidiae* but the GP-PVPP had no antimicrobial activity. The high affinity of the identified PCs from GPs on PVPP polymer could allow the design of new processes and by-products for the food or cosmeceutical industry, promoting a circular economy by reducing and reusing wastes (GPs and PVPP) and organic solvents.

## 1. Introduction

Grape pomace (GP) is obtained after the winemaking process, which contains a large number of polyphenolic compounds (PCs) with antioxidant properties [1]. Around 6000 tons of this waste is produced in the world [2], which is not efficiently reused as a by-product, losing a great variety of phenolic compounds present in this potential raw material. Therefore, some efforts for its reutilization as a food-fortifying ingredient have been performed [3]. Between 60 and 70% of the total PC content from the grape is retained in the GP containing a great number of bioactive antioxidants [4]. Among the PCs present in GP, anthocyanins, such as malvidin, delphinidin, cyanidin, and petunidin [2,5], are characteristic red pigments that are expressed during fruit ripening. They are highly susceptible to chemical transformations due to the action of external agents, such as light, temperature, oxygen, and pH. Stilbenes (phytoalexins) are also present in GP. The main stilbene found in grapes is resveratrol [6]. Flavonols, such as kaempferol, quercetin, and myricetin, are present [7]. Finally, phenolic acids, mainly chlorogenic acid, gallic acid, and coumaric acid, are present in a GP extract [8,9,10]. In addition to antioxidant activity, these compounds have beneficial effects on humans, including from GP waste [3,11].

The antibacterial activity of the GP extract depends on the initial composition of the GP (seed, skin, stems, or their combination), wine process, and grape cultivar. Thus, the chemical profiles of the extracts vary and, therefore, their bioactivity. In this context, Tseng et al. [12] showed that GP composed of seed and skin has a higher antioxidant capacity and antibacterial activity against *Listeria innocua* ATCC 51,142 and *Escherichia coli* ATCC 25,922 in comparison to only skin GP. Gerardi et al. [8] showed that whole GP and only skin GP had antibacterial activity against *Staphylococcus aureus* and *Enterococcus faecalis*, with higher sensitivity to *Pseudomonas* spp. In addition, the red skin GP had higher antibacterial activity in comparison to white, due to its phenolic acids and anthocyanins composition. Ghendov-Mosanu et al. [9] describe the extraction of flavonoids, flavones, hydroxybenzoic acid derivatives, hydroxycinic acid derivatives, and ferulic acid methyl ester from the *Merlot* GP with higher antimicrobial activity against Gram-positive than Gram-negative. In addition, the GP extract from different grape cultivars of Turkey showed antimicrobial activity on food (beef patty and foodborne). The main compounds were phenolic acids, flavonols, and Stilbenes [13].

The purification of PCs from different matrices is performed by successive steps: extractions, and isolation/concentration. The isolation step is mainly made by chromatographic separation on an open column mode, among other preparative modes, such as HPLC and TLC. Different mechanisms of separation are used by varying the stationary phases (polymers, ion exchange, and hydrophobic phases) [14], or by countercurrent (liquid–liquid) chromatography [15]. Membrane purification has also been employed but membrane fouling and reduced selectivity restrict their use [16].

The polyvinylpolypyrrolidone (PVPP) polymer has gained attention in the purification of PCs due to its use at an industrial level for the clarification and stabilization of beer, wine, and juices [17]. However, it is not reused in these industries losing valuable PCs [18]. The PVPP is an inert and insoluble cross-linked polymer that adsorbs PCs mainly through hydrogen bonding and hydrophobic interaction. The PCs with the highest affinity are quercetin, catechin, epicatechin, caffeic acid, and gallic acid [19,20]. The affinity and selectivity of PCs for binding to PVPP increase as the molecule has more aromatic rings and hydroxyl groups [21,22]. The PCs adsorbed in this polymer can be desorbed to favor the reuse of PVPP [23]. Despite these advantages, the purification of PCs from GP is not so explored, and the adsorbed compounds depend on the GP composition (skin, seeds, stems) and grape cultivar.

The PC adsorption from fruits or agro-industrial by-products onto PVPP has not been extensively reported [18]. Therefore, this research seeks to reuse the GP waste from the winemaking process for the obtention of a PC-rich extract adsorbed on PVPP to use as an antimicrobial material. For this, *Carménère*, *Cabernet Sauvignon*, and *Merlot* cultivars, which has the highest demand in Chile for winemaking (Chilean Agricultural and Livestock Service, SAG, 2016), were used to obtain the GPs. The extraction and composition of PCs from the GPs ethanolic extracts and their adsorption on PVPP columns were evaluated. In addition, the potential antimicrobial activity of the GP extract and its composite (PC–PVPP) was also evaluated. In this way, it would be possible to value a by-product rich in PC and promote the circular economy by reducing waste (GP and PVPP).

## 2. Materials and Methods

### 2.1. Reagents, Solvents, and Standards

For the TPC analysis and antioxidant capacity, all chemicals used were of analytical grade. Folin–Ciocalteu phenol reagents, sodium carbonate, gallic acid, potassium dihydrogen phosphate, quercetin, and polyvinylpolypyrrolidone were acquired from Merck (Darmstadt, Germany). Fluorescein sodium salt, 2,2’azobis(2-methylpropionamidine) dihydrochloride (AAPH), (±)–6-Hydroxy-2,5,7,8-tetramethylchromane-2-carboxylic acid (Trolox), sodium phosphate dibasic, pelargonidin chloride and (±)-catechin were acquired from Sigma-Aldrich (St. Louis, MO, USA).

For analyzes performed using RP-UHPLC, all reagents were HPLC grade. Acetonitrile, formic acid, ethanol, and water were acquired from Merck.

### 2.2. Plant Material

*Carménère*, *Cabernet Sauvignon,* and *Merlot* red GPs were obtained from the San Pedro wine cellar (Molina, Maule Region, Chile) after the winemaking process. They were stored at −20 °C in airtight containers until use.

### 2.3. Extractions of Polyphenols

The extraction was performed according to the procedure described by Ribeiro et al. [24] with some modifications. Briefly, two masses of GP (0.5 g and 1 g) were macerated in 75 mL of ethanol/water (60:40 *v*/*v*) as the extraction solvent. Then, different maceration times (1–48 h) with and without agitation were performed. All experiments were extracted at 21 °C. Three replicates for each experiment were performed. The mass-volume ratio that presented the highest PC content was used to perform the adsorption process with PVPP.

### 2.4. Determination of Total Phenolic Compounds (TPC) and Antioxidant Capacity

Folin–Ciocalteu method. The equivalent content of total phenolic compounds was determined through the Folin–Ciocalteu method according to Song et al. [25] with some modifications. Briefly, Folin–Ciocalteu reagent, 20% (*m*/*v*) sodium carbonate solution was used. The absorbance of the samples was measured with a Synergy HTX Multi-mode reader (BioTek Instruments, Winooski, VT, USA) at a wavelength of 765 nm. The results were expressed as gallic acid equivalent (GAE).

Oxygen radical absorption capacity (ORAC-FL). The methodology of Folch-Cano et al. [26] and Ou et al. [27] was used with some modifications. The reaction mixture was composed of 75 mM of sodium phosphate buffer (pH 7.4), 70 nM of fluorescein, 9 mM AAPH, and 20 µL of GP extract. The Trolox (6–72 μM) compound was used as the standard for the calibration curve. The reaction was incubated for 15 min at 37 °C in the microplate reader and the decrease in fluorescence over time was registered (Ex: 495 nm and Em: 515 nm) with the GEN5 software. The ORAC index (µmol of Trolox/g of GP) was obtained by graphic interpolation of the area under the curve of the sample in the standard curve using OriginPro 8.5.1.315 software (Northampton, MA, USA).

### 2.5. Adsorption of TPC from GP Extract in Polyvinylpolypyrrolidone (PVPP)

Syringes, to build the adsorption columns, were used. Each column was filled with glass wool on the tip and 200 mg of PVPP. This column was conditioned with ethanol:water (60:40, *v*:*v*). GP extract was passed through this column during twelve successive processes (3 mL each time), to evaluate the adsorption of the PCs in the PVPP. The column was connected to a manifold and a vacuum pump with a controlled pressure (−50 KPa). The TPC, antioxidant capacity and UHPLC-DAD-MS analysis were performed to evaluate the PCs in the extracts before and after passing through the PVPP column. The difference between the extract without passing through the PVPP and the one that passed through the PVPP was quantified as the PC retained (%) in the column.

### 2.6. Identification of PCs before and after Adsorption in PVPP

The polyphenolic compounds were identified and quantified by UHPLC-DAD-MS (Shimadzu UHPLC LCMS-8030, Kyoto, Kansai Region, Japan). This equipment consists of a quaternary LC-30AD pump, DGU-20A5R degasser unit, CTO-20AC oven, SIL-30AC auto-sampler, CBM-20A controller system, and UV-Vis diode array spectrophotometer (model SPD-M20A), coupled in tandem with a Mass Spectrometer (MS). Instrument control and data processing were done using LabSolutions software (version 5.86).

Anthocyanins, flavonols, and flavan-3-ols were separated in a shim-pack XR-ODS III column (particle size of 2.2 µm, 2.0 mm i.d, and 200 mm length, Shimadzu, Kyoto, Kansai Region, Japan). A shim-pack GVP-ODS precolumn (2.0 mm i.d. × 5 mm length, Shimadzu) was used. The flow rate and column temperature were set at 0.3 mL/min and 30 °C, respectively. The injection volume was 30 µL. The mobile phase consisted of 0.1% (*v*/*v*) of formic acid in water (A) and acetonitrile (B). The gradient program was as followed: 15 min from 10% to 20% of B, 6 min at 20% of B, 5 min from 20% to 27% of B, 10 min at 27%, 1 min from 27% to 100%. The diode array detector was set from 190 to 700 nm.

Mass spectrometry analysis to identify the polyphenols was performed. The electrospray (ESI) source block and desolvation temperature were set at 400 and 250 °C, respectively. Drying and nebulizing gas flow rates were set at 15 and 3 L/min, respectively. Interface voltage and interface current was set at 3.5 kV and 0.2 mA, respectively. The compound identification was performed by a Q1 ion scan (100 to 1.700 *m*/*z*) in positive and negative ESI mode with the synchronized survey product ion scan (threshold: 10.000 cps, scan: 100 to 500 *m*/*z*, collision energy (CE): −35 V).

Quantification of the identified compounds was done by RP-UHPLC-DAD (Shimadzu UHPLC LCMS-8030, Kyoto, Kansai Region, Japan) with the same chromatographic elution gradient. An external calibration curve was performed with the standard pelargonidin for anthocyanin (520 nm), quercetin for flavonols (320 nm), and catechin for flavanols (280 nm).

### 2.7. Bacterial Strains, Culture Media, and Growth Conditions

Analytical grade culture media were of analytical grade. Muller-Hinton agar and Brain heart infusion were acquired from Merck. *Bacillus subtilis*, *Bacillus cereus*, *Escherichia coli*, *Staphylococcus aureus*, *Salmonella enteritidis*, *Pseudomonas syringae* pv. *actinidiae* and *Listeria monocytogenes* were acquired from Microbiology and Mycology Laboratory from the Agricultural Engineering Faculty (Universidad de Concepción).

Antimicrobial inhibition zone test. The test cultures were grown separately in brain heart broth (BHI) for 22 ± 2 h at 37 °C. The surface seeding method with the Müller Hinton agar base was used by inoculating ~1 × 10^8^ CFU/mL. After sowing, sensi-discs agar (diameter of 6 mm) was deposited on the surface of the agar to evaluate the antibacterial activity of GP extracts. Three aliquots (5, 10, and 20 μL) were tested with an initial concentration of 10 mg mL^−1^ for the different tested organisms. Distilled water, quercetin, and streptomycin (10 mg/mL) were used as the negative, antioxidant, and positive controls, respectively. The plates were incubated at 37 °C for 16–18 h, after which the inhibition zones (mm) were measured to determine the antibacterial activity [28]. The composite GP-PVPP was also evaluated for antibacterial activity. For this, 10 mg of the GP-PVPP was added to the plate.

### 2.8. Statistical Analysis

Statistical significance variation between results was performed by analysis of variance (ANOVA) with Tukey’s multi-comparison test. Differences were considered statistically significant at a *p* < 0.05.

## 3. Results

### 3.1. Polyphenol Extraction

**Figure 1:**
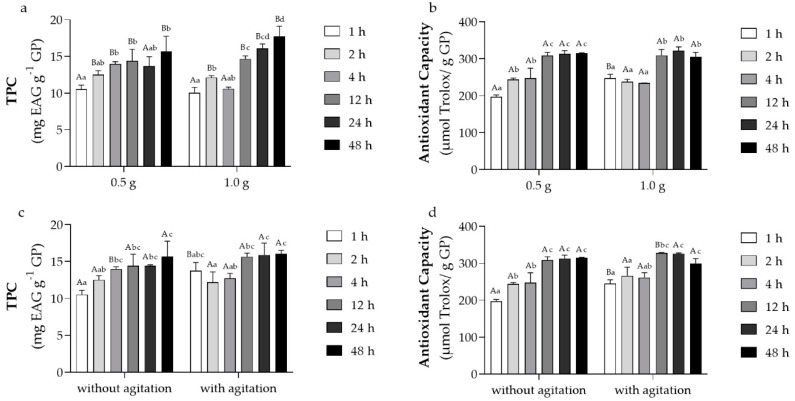
TPC and antioxidant capacity (ORAC-FL) of *Carménère* GP extract at different maceration times. Effects of GP masses (**a**,**b**) and agitation (**c**,**d**) during the extraction are presented. Means and standard deviations are shown. Different capital letters indicate significant differences (*p* > 0.05) in the TPC or antioxidant capacity between masses or agitation at the same time. Different lowercase letters indicate differences (*p* > 0.05) in the TPC and antioxidant capacity between different times at the same mass or agitation condition.

The extraction process was evaluated to achieve the highest content of PC by evaluating different amounts of *Carménère* GP mass, extraction time, and agitation during the maceration. The extraction of PC from *Carménère* GP is shown in Figure 1. We observe that the extraction of PC depends on the maceration time and the amount of GP mass, reaching the higher TPC (Figure 1a) and antioxidant activity (Figure 1b) at an extraction time of 12 h and 0.5 g. Nevertheless, no significant difference above this extraction time was achieved. The agitation at different extraction times did not show an important increase in TPC (Figure 1c) or antioxidant capacity (Figure 1d). Therefore, based on the comparison of TPC and antioxidant capacity results from the GP extracts, the selected parameters of extraction were 0.5 g of GP during 12 h of maceration without agitation.

### 3.2. TPC and Antioxidant Capacity of GP Extract

The GP extracts from *Carménère*, *Cabernet Sauvignon*, and *Merlot* were obtained with the previously selected method (Section 2.1) and their TPC and antioxidant capacity were determined. The TPC for *Carménère* was 60.86 ± 7.49 mg GAE g^−1^ of GP extract, for *Cabernet Sauvignon* was 57.03 ± 4.78 mg GAE g^−1^ of GP extract and for *Merlot* 70.36 ± 1.75 mg GAE g^−1^ of GP extract. The antioxidant capacity (ORAC-FL) was 1050.87 ± 76.69, 821.48 ± 108.30, and 1255.52 ± 137.60 μmol Trolox g^−1^ GP extract, respectively.

### 3.3. PC Composition of GP Extract

The (poly)phenolic composition of each GP extract was determined by RP-UHPLC-DAD-ESI-MS/MS analysis (Table 1). Six anthocyanin compounds were identified, where malvidin derivates (malvidin-3-O-(6-acetyl)-glucoside, malvidin-3-O-glucoside, malvidin-3-O-(6-caffeoyl)-glucoside, and malvidin-3-O-(6-coumaroyl)-glucoside) are common to all samples. On the other hand, peonidin-3-O-glucoside and delphinidin-3-O-(6-caffeoyl)-glucoside were not identified in *Merlot* and *Cabernet Sauvignon* GP extracts, respectively. For flavonols, the myricetin-3-O-galactoside, quercetin-3-O-glucoside, and quercetin were in the three GP extracts, while myricetin-3-O-glucuronide was only in GP of *Cabernet Sauvignon* and myricetin-3-O-glucoside in GP of *Carménère* and *Merlot*. On the other hand, the identified flavan-3-ol were catechin and epicatechin for all samples.

### 3.4. Phenolic Compounds Quantification in GP Extracts

The quantification of the PC in the GP extracts is presented in Table 2. The higher anthocyanin concentration was extracted from *Carménère* GP, followed by *Merlot*, and *Cabernet Sauvignon* GPs. The anthocyanins with higher concentration were malvidin-3-O-(6-coumaroyl)-glucose for *Carménère* and *Merlot*. The higher flavanols concentration were extracted from *Carménère* GP, followed by *Merlot,* and *Cabernet Sauvignon* GPs. Catechin flavan-3-ol had a higher concentration in *Carménère* and *Merlot*. On the other hand, the GP extract of *Cabernet Sauvignon* showed low levels of flavanols (<LOD), especially catechin, due to the low amount of seed in the GP, which is the main source of this compound [1]. Total flavonols were found in a higher proportion in *Carménère*, followed by *Cabernet Sauvignon* and *Merlot*. Variations of GP extract compositions were observed especially for flavonols.

### 3.5. Adsorption of TPC on PVPP

**Figure 2:**
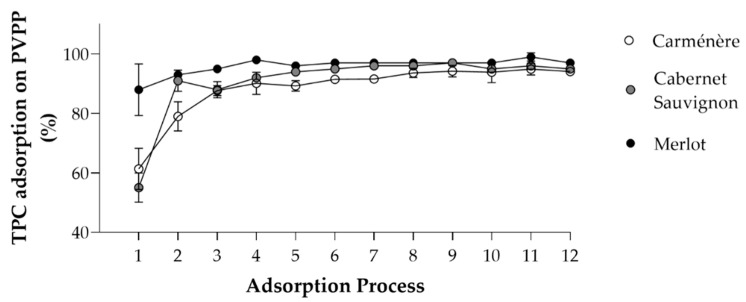
Percentage of TPC from GP extract adsorbed on PVPP. *Carménère* (white), *Cabernet Sauvignon* (grey), *Merlot* (black) extracts. Each adsorption process (1–12) was performed with 3 mL of GP extract. Means and standard deviations are presented for each treatment.

The TPC adsorbed on PVPP after the twelve adsorption processes were equivalent to 3.6 mg GAE for *Carménère*, 3.4 mg GAE for *Cabernet Sauvignon*, and 5.11 mg GAE for *Merlot*. The adsorption profile (Figure 2, Figure S1) showed that the total retention of TPC increased until the saturation of PVPP, which was in the range of 93–97 mg GAE for the three GP extract. After the third loading of GP extract on the columns, the PVPP was saturated. The *Carménère* GP extract had the lowest PC adsorption per charge on the polymer and the *Merlot* GP extract had the highest. These results showed the capacity of PVPP columns to concentrate PC.

In addition, the GP extract without the adsorbed PCs did not show antioxidant capacity (data not shown) indicating selective retention of PC and the presence of interferents that contribute to an overestimation of the TPC by the Folin–Ciocalteu method, such as sulfite and sugars [29]. Overall, it was possible to concentrate the PC in the PVPP during several loading cycles of GP extracts until saturation of the polymer.

### 3.6. Composition of PCs Absorbed in PVPP

**Figure 3:**
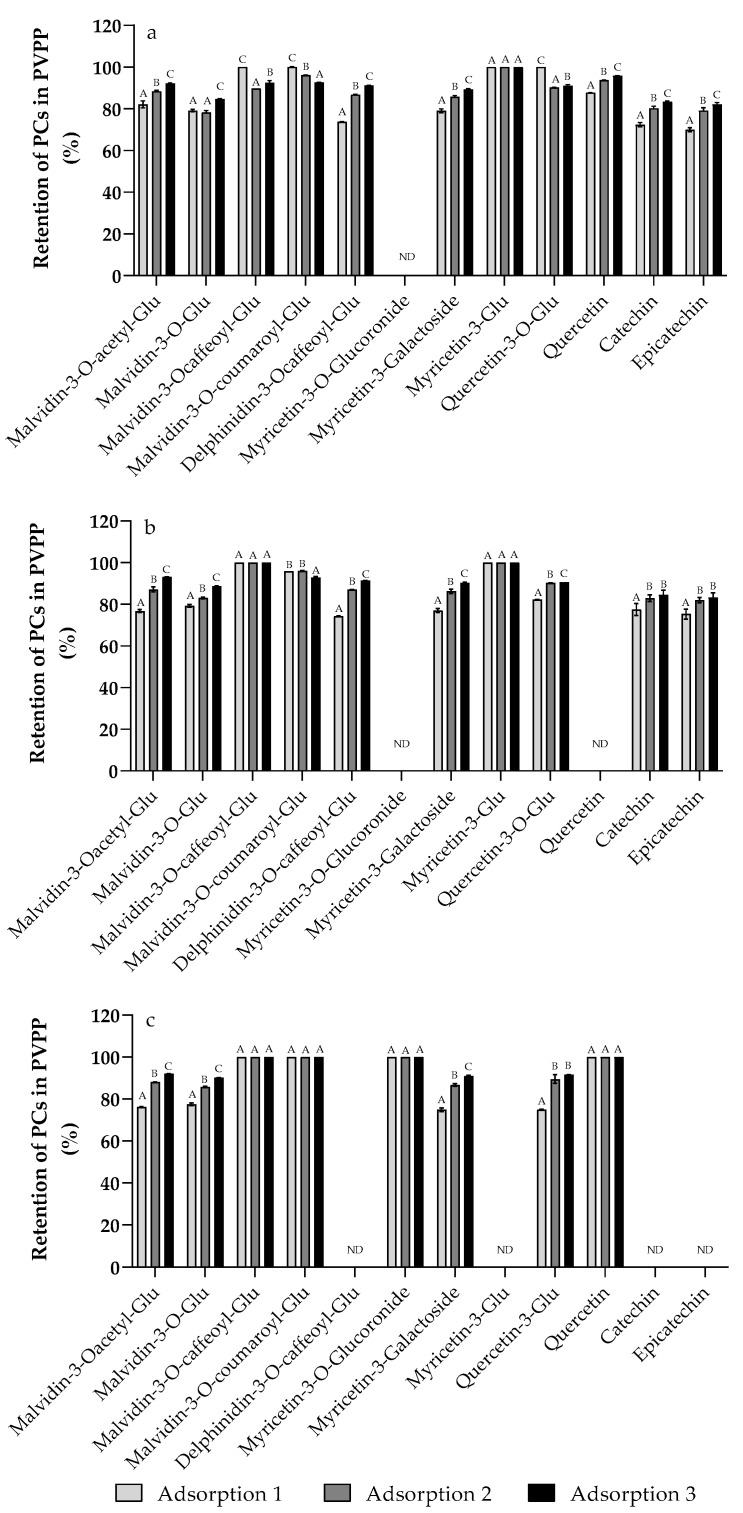
Percentage of PC adsorbed on PVPP for each quantified compound from *Carménère* (**a**), *Merlot* (**b**), and *Cabernet Sauvignon* (**c**) GPs extracts. Adsorptions 1, 2, and 3 correspond to consecutive adsorption processes from 1 to 4, 5 to 8, and 9 to 12, respectively. Different capital letters indicate significant differences (*p* > 0.05) in the TPC or antioxidant capacity between masses or agitation at the same time. ND: Not detected (<LOD).

The GP extracts were loaded in the PVPP column, therefore, the number of PCs loaded in this column was quantified (Table 3, Figure S1). The adsorption capacity was evaluated for each polyphenol adsorbed (Figure 3). The higher percentage of the PC adsorbed in the polymer were flavonols, followed by flavan-3-ols and anthocyanins. The behavior of the compounds analyzed individually is similar to that observed in the adsorption determined by the Folin–Ciocalteu method. Among the compounds that showed lower adsorption, we found malvidin-3-O-glucoside for the three GPs analyzed. In contrast, 100% adsorption of malvidin-3-O-(6-caffeoyl)-glucoside, malvidin-3-O-(6-coumaroyl)-glucoside, myricetin-3-O-glucoside, and quercetin-3-O-glucoside was observed for the extracts of *Cabernet Sauvignon*. Similar results were observed in the adsorption of compounds from *Merlot*, which also has complete adsorption of myricetin-3-glucoside.

After twelve adsorption processes, PVPP continues to retain compounds, depending on their affinity, showing the selectivity and affinity to phenolic compounds with high antioxidant capacity present in the GP extract.

### 3.7. Bacterial Inhibition Activity

The antibacterial activity of the GP extract and its PVPP composite was evaluated on four Gram-positive and three Gram-negative bacteria from clinical and agronomic relevance (Table S1, Figure S2). Within the Gram-positive bacteria (*B. cereus*, *B. subtilis*, *S. aureus,* and *L. monocytogenes*), the highest amount of GP extract from *Carménère* and *Merlot* was able to inhibit the growth of *B. cereus*. The other Gram-positive bacteria were not affected by the GP extracts. Quercetin control had only inhibition effect on *S. aureus*. On the other hand, within the Gram-negative bacteria (*P. syringae* pv. *actinidiae*, *E. coli,* and *Salmonella* sp.), only *P. syringae* pv. *actinidiae* was inhibited by *Carménère* and *Cabernet Sauvignon* GP extracts. The quercetin control showed a growth inhibition effect on this bacterium. None of the other bacteria were affected by the GP extracts.

The bacterial Inhibition effect of the PCs from the GP retained on the PVPP polymer was also evaluated. The results showed no inhibitory activity against the tested bacteria, due to a low or no diffusion of the retained compound on the PVPP polymer to the agar medium, indicating a strong interaction between them. Nevertheless, the PCs can be desorbed from PVPP [23] to formulate antibacterial products based on the reuse of GP and PVPP.

## 4. Discussion

Grape pomace (GP) is a by-product resulting from the winemaking process. The extraction of valuable bioactive compounds from GP is possible and has been slightly explored. These GP bioactive compounds can be retained in the well-known PVPP to favor their stability and to develop functional materials. Therefore, *Carménère*, *Cabernet Sauvignon*, and *Merlot* GPs from the Chilean wine industry were used and their (poly)phenolic composition was determined. Phenolic acids have high retention on PVPP polymer as reported by other authors [21,29]. Thus, we evaluated the anthocyanins, flavonols, and flavanols adsorption on PVPP, which are less reported, especially from the GP of our region. In addition, the GP extract and GP-PVPP antimicrobial activity were analyzed.

In this study, we used maceration (12 h and 0.5 g mass) as an extraction technique due to a lower cost of implementation making it possible for industrial scaling, in comparison to other technologies. Overall, *Merlot* presented higher TPC and antioxidant capacity, followed by *Carménère* and *Cabernet Sauvignon*. The difference between these values was attributed to several factors, such as the winemaking process, seed size, genetics, environment, and agronomic management [30]. The TPC obtained for *Cabernet Sauvignon* and *Merlot* is close to those reported by Lorrain et al. [31] who had values of 68.6 and 77.1 mg GAE g^−1^ DM, respectively. Moreover, Rockenbach et al. [30] presented similar results in the GP from Brazilian *Cabernet Sauvignon and Merlot* (74.75 ± 2.22 and 46.23 ± 1.63 mg GAE g^−1^ DM). In these results, a higher TPC exhibited a greater antioxidant capacity due to their polyphenolic composition. In addition, the TPCs and antioxidant capacity in the GP extracts were higher than reported by Mejia et al. [32], Nayak et al. [33], and Milincic et al. [34], which performed extractions using aqueous assisted with enzymes/ultrasound, with water, and methanol, respectively. We used ethanol as the extraction solvent, which is allowed for food development.

The main flavonoids identified in the extracts were anthocyanins, flavonols, and flavan-3-ol, as described by Beres et al. [2]. Total anthocyanin, flavonols, and flavanols were higher in the *Carménère* GP extract (Table 2) than in *Merlot* and *Cabernet Sauvignon* showing a clear difference between the cultivars. This is explained by the type of cultivar, the winemaking process, and GP composition (seed, skin, stems, or their combination). In addition, our results are according to Caldas et al. [35] and Ribeiro et al. [24], who reported similar compounds on *Merlot*, *Cabernet Sauvignon*, and red grapes varieties. The cyanidin and petunidin derivates were not identified in our GP extracts. Similarly, Huaman-Castilla et al. [36] reported anthocyanin derivates from fresh *Carménère* grape skins, being malvidin, cyanidin, petunidin, delphinidin, and peonidin the most abundant. For flavonols, our results are consistent with Aguilar et al. [5], Lorrain et al. [31], and Caldas et al. [35] who reported that in grape juice the catechin and epicatechin are present, in addition to the aforementioned compounds. In general, the content of anthocyanins, flavonols, and flavan-3-ol are similar among the GP extracts. However, a high number of these compounds are present in red wine, such as petunidin, pyrano peonidin, and their derivates [37], indicating that were removed from the GP during the winemaking process. In the same way, PVPP used in the white wine process is able to extract polyphenols, mainly proanthocyanidins and hydroxycinnamic acids, with strong antioxidant activity favoring the reuse of this polymer and valorization of the extracted polyphenols [38].

The formation of composites between this valuable PC from the GP extracts and PVPP polymer could increase the commercial applications of the GPs by generating bioactive materials with PVPP, as a purification step or as a stabilizing agent. In our study, this polymer showed high adsorption capacity for PCs from the three GP extracts, and the adsorption differences obtained between them are attributed to its initial composition.

The retention of PCs is in agreement with the adsorption of pure PCs on PVPP, where the presence of hydroxyl groups, hydrophobic interaction, H-bonds, and Van der Waals interaction favors their retention [21,39]. In this context, Duran-Lara et al. [19] and Magalhaes et al. [20] described the adsorption of 100% of quercetin and 60% of catechin on the PVPP polymer. In our study, the differences in adsorption between flavonols and flavan-3-ols can be attributed to the spatial distribution of hydroxyl groups that interact through hydrogen bonding with PVPP [17,21,40]. Concerning anthocyanins, the low retention can be attributed to the lower content of hydroxyl groups and the presence of methyl groups in their chemical structure. This fact explains the low adsorption capacity of PC from *Carménère* extracts which showed the highest amount of anthocyanins compared to *Merlot* and *Cabernet Sauvignon* extracts.

Other polymers have been used to recover or purify PCs from different matrices and can be reviewed by Soto et al. [41]. Nevertheless, the adsorption of PCs from GPs extracts on different materials is scarce. Kammerer et al. [42] used Amberlite XAD resins to retain anthocyanin from the *Cabernet Mitos* variety GP (water extract) obtaining recoveries >96%. In addition, natural polymers such as pectin and gelatin improved the stability of the PCs extracted from GPs [43], and chitosan-based films with GP extracts have also been developed [44]. Synthetic polymers have been used to retain flavan-3-ol extracted from the variety *Tempranillo* GPs and for ultrafiltration to fractionate the PCs, allowing recoveries higher than 96% [45]. The liquid–liquid extraction using an aqueous two-phase system (ATPS) composed of acetonitrile/PVP/water has also been proposed for the partition of polyphenols using a mixture of pure compounds [46]. The ATPS is a promising alternative to separate polyphenol extracts but further studies must be done to favor the extraction of these compounds from GP residues. On the other hand, the adsorption on PVPP has a series of advantages, such as a high retention capacity and stability, it is currently used in the wine process, and can be reusable if the PCs are desorbed.

The antibacterial activity of PCs has been extensively demonstrated [10,28,47,48,49,50]. We observed inhibition of *B. cereus* by the GP extract from *Carménère* and *Merlot*. This bacterium is found mainly in soil and its toxins can contaminate food such as rice grain causing diarrheal or emetic (nausea and vomiting) in foodborne [51,52]. Thus, these GP extracts can be used to prevent *B. cereus* in an agronomic or clinical stage. On the other hand, the *P. syringae* pv. *actinidiae* bacterium was inhibited by *Carménère* and *Cabernet Sauvignon* GP extracts. This bacterium is a phytopathogen that causes bacterial canker infection on leaves or trunks of kiwifruit trees leading to important commercial losses in the industry [53].

According to the composition of the GP extracts, quercetin and myricetin in *Carménère* and *Cabernet Sauvignon* GP extracts, are known for their antibacterial effects due to a greater number of hydroxyl groups in their structure. On the other hand, extracts with a higher concentration of anthocyanins show a drastic decrease in their antibacterial activity, due to a lower content of hydroxyl groups and the presence of methoxyl groups in their structure [13]. Three mechanisms of action for the antioxidant compounds with inhibitory effects against bacteria are reported: inhibition of the biosynthesis of nucleic acids or cell walls; damage of the integrity of membranes and/or interference with the great variety of essential metabolic processes [54].

In general, the characterization of the GP extract showed a high diversity on PC, which is a waste after the winemaking process. In addition, its adsorption on PVPP favors PC concentration and desorption strategies during the wine fining have been reported to reuse PVPP [23]. This research opens the possibility for the revalorization of GP and the generation of high-value products such as antibacterial agents.

## 5. Conclusions

The GP obtained from the winemaking process is not efficiently reused as a by-product. In this study, we showed that it is possible to obtain a high content of PCs from this waste. Likewise, it was shown that these compounds are retained in PVPP in a high percentage during successive adsorption cycles. Regarding the antibacterial activities of the extracts, the *Carménère* and *Cabernet Sauvignon* strains showed growth inhibition against the bacteria *B. cereus* and *P. syringae* pv. *actinidiae*, which affect humans and the production of several agricultural foods. Nevertheless, the determination of phenolic acids and the minimum inhibitory concentration values against these bacteria must be further evaluated. The PCs of the GP extract adsorbed in PVPP (GP-PVPP) had no inhibition activity on the tested bacteria, but this polymer was able to remove the PC interferents from the GP extract. The desorption process of these compounds must be evaluated to generate a PC-rich solution with potential antimicrobial and antioxidant activities for future applications in food or cosmeceutical industries.

## Figures and Tables

**Table 1:** Chemical characterization by UHPLC-DAD-ESI-MS/MS of polyphenols in GP extract. GP Origin: *Carménère* (a), *Merlot* (b), *Cabernet Sauvignon* (c).

Anthocyanins	tR (min)	Ion Mode	Precursor Ion (*m*/*z*)	Product Ion (*m*/*z*)	GP Origin
Malvidin-3-O-(6-acetyl)-glucoside	16.52	+	535	331/315	a,b,c
Malvidin-3-O-glucoside	9.00	+	493	331/315/287	a,b,c
Malvidin-3-O-(6-caffeoyl)-glucoside	19.73	+	655	331	a,b,c
Malvidin-3-O-(6-coumaroyl)-glucoside	23.52	+	639	331	a,b,c
Peonidin 3-O-glucoside	8.36	+	463	301/286/258	a,c
Delphinidin-3-O-(6-caffeoyl)-glucoside	15.25	+	627	303	a,b
Flavonols					
Myricetin-3-O-glucuronide	16.55	+	495	319/153/126/103	c
Myricetin-3-galactoside	15.37	+	481	319/245/165	a,b,c
Myricetin-3-glucoside	20.22	+	481	319/305/254	a,b
Quercetin-3-O-glucoside	19.13	+	465	303/229/153	a,b,c
Quercetin	34.30	+	303	137/121/109	a,b,c
Flavan-3-ols					
Catechin	7.99	+	291	123/139/161	a,b,c
Epicatechin	11.48	+	291	123/139/161	a,b,c

**Table 2:** The concentration of polyphenols in GP extracts. Means with a common capital letter are not significantly different (*p* > 0.05). Capital letters indicate significant differences between a compound in different extracts. Lowercase letters indicate significant differences between total values in different extracts. ND: Not detected (<LOD).

Compound	GP Extract (µg Equivalent/g Extract)		
Anthocyanin	*Carménère*	*Merlot*	*Cabernet Sauvignon*
Malvidin-3-O-(6-acetyl)-glucoside	4.24 ± 0.06 **^A^**	2.47 ± 0.03 **^B^**	2.54 ± 0.03 **^B^**
Malvidin-3-O-glucoside	11.24 ± 0.74 **^A^**	5.05 ± 0.03 **^B^**	3.38 ± 0.02 **^C^**
Malvidin-3-O-(6-caffeoyl)-glucoside	3.14 ± 0.01 **^A^**	2.67 ± 0.02 **^B^**	2.59 ± 0.15 **^B^**
Malvidin-3-O-(6-coumaroyl)-glucoside	13.95 ± 0.25 **^A^**	11.91 ± 0.10 **^B^**	2.43 ± 0.16 **^C^**
Delphinidin-3-O-(caffeoyl)-glucoside	2.56 ± 0.08 **^A^**	2.00 ± 0.03 **^B^**	ND
Total Anthocyanin	35.13 ± 1.13 ^a^	24.10 ± 1.13 ^b^	10.94 ± 0.36 ^c^
Flavonols			
Myricetin-3-O-glucuronide	ND	ND	0.56 ± 0.09 **^A^**
Myricetin-3-O-galactoside	0.91 ± 0.07 **^A^**	0.55 ± 0.06 **^B^**	0.58 ± 0.09 **^B^**
Myricetin-3-O-glucoside	0.80 ± 0.01 **^A^**	0.75 ± 0.04 **^B^**	n/d
Quercetin-3-O-glucoside	0.68 ± 0.01 **^A^**	0.58 ± 0.01 **^B^**	0.54 ± 0.06 **^B^**
Quercetin	1.17 ± 0.05 **^A^**	n/d	0.49 ± 0.04 **^B^**
Total Flavonols	3.56 ± 0.14 ^a^	1.88 ± 0.11 ^b^	2.17 ± 0.28 ^b^
Flavanols			
Catechin	4.68 ± 0.10 **^A^**	4.12 ± 0.03 **^B^**	ND
Epicatechin	2.64 ± 0.03 **^A^**	2.58 ± 0.04 **^A^**	ND
Total Flavanol	7.32 ± 0.13 ^a^	6.70 ± 0.07 ^b^	ND

**Table 3:** PC content from the extract loaded in the PVPP. Quantification by RP-UHPLC-DAD.

Grape Pomace Extract	Loaded Mass (µg Equivalent)								
	*Carménère*			*Merlot*			*Cabernet Sauvignon*		
Anthocyanins	1	2	3	1	2	3	1	2	3
Malvidin-3-O-(6-acetyl)-glucoside	75.3 ± 4.5 ^a^	150.5 ± 9.1 ^a^	225.8 ± 13.6 ^a^	57.0 ± 4.1 ^b^	114.0 ± 8.2 ^b^	171.0 ± 12.4 ^b^	48.9 ± 2.4 ^c^	97.8 ± 4.8 ^c^	146.7 ± 7.2 ^b^
Malvidin-3-O-glucoside	189.0 ± 5.1 ^a^	378.1 ± 10.2 ^a^	567.1 ± 15.4 ^a^	117.4 ± 4.9 ^b^	234.8 ± 9.8 ^b^	352.2 ± 14.7 ^b^	63.9 ± 3.1 ^c^	127.8 ± 6.4 ^c^	191.8 ± 9.4 ^c^
Malvidin-3-O-(6-caffeoyl)-glucoside	56.4 ± 3.4 ^a^	112.8 ± 6.9 ^a^	169.3 ± 10.3 ^ab^	62.9 ± 4.3 ^a^	125.8 ± 8.7 ^a^	188.7 ± 13.0 ^a^	46.3 ± 4.3 ^b^	92.7 ± 8.5 ^b^	139.0 ± 12.8 ^b^
Malvidin-3-O-(6-coumaroyl)-glucoside	246.5 ± 20.2 ^a^	493.0 ± 40.5 ^a^	739.6 ± 60.7^a^	276.6 ± 18.1 ^a^	553.3 ± 36.2 ^a^	829.9 ± 54.3 ^a^	49.1 ± 5.2 ^b^	98.2 ± 10.3 ^b^	147.3 ± 15.5 ^b^
Delphinidin-3-O-(6-caffeoyl)-glucoside	47.5 ± 3.1 ^a^	95.1 ± 6.2 ^a^	142.6 ± 9.3^a^	46.0 ± 2.6 ^a^	92.0 ± 5.3 ^a^	137.9 ± 7.9 ^a^	-	-	-
Flavonols									
Myricetin-3-O-glucuronide	-	-	-	-	-	-	9.0 ± 0.1	17.9 ± 0.1	26.9 ± 0.2
Myricetin-3-O-galactoside	15.1 ± 0.9 ^a^	30.2 ± 1.8 ^a^	45.2 ± 2.7 ^a^	11.6 ± 0.5 ^b^	23.2 ± 0.9 ^b^	34.8 ± 1.4 ^b^	9.44 ± 0.01 ^c^	18.89 ± 0.02 ^c^	28.33 ± 0.04 ^c^
Myricetin-3-O-glucoside	14.6 ± 0.8 ^b^	29.2 ± 1.6 ^b^	43.7 ± 2.3 ^b^	18.3 ± 0.7 ^a^	36.7 ± 1.4 ^a^	55.0 ± 2.1 ^c^	-	-	-
Quercetin-3-O-glucoside	12.2 ± 0.7 ^a^	24.3 ± 1.3 ^a^	36.5 ± 2.0 ^a^	13.2 ± 0.5 ^a^	26.3 ± 0.9 ^a^	39.5 ± 1.5 ^a^	9.09 ± 0.03 ^b^	18.2 ± 0.1 ^b^	27.3 ± 0.1 ^b^
Quercetin	20.3 ± 1.0 ^a^	40.6 ± 1.9 ^a^	61.0 ± 2.9 ^a^	-	-	-	9.9 ± 0.1 ^b^	19.9 ± 0.1 ^b^	29.8 ± 0.1 ^b^
Flavan-3-ols									
Catechin	82.4 ± 3.9 ^b^	164.7 ± 7.8 ^b^	247.1 ± 11.7 ^b^	95.8 ± 4.0 ^a^	191.6 ± 8.0 ^a^	287.4 ± 12.0 ^a^	-	-	-
Epicatechin	47.9 ± 2.7 ^b^	95.9 ± 5.3 ^b^	143.8 ± 8.0 ^b^	59.4 ± 3.7 ^a^	118.8 ± 7.5 ^a^	178.2 ± 11.2 ^a^	-	-	-

1: Total equivalent mass loaded during the adsorption process 1 to 4; 2: Total equivalent mass loaded during the adsorption 5 to 8; and 3: Total equivalent mass loaded during the adsorption 9 to 12. (−): Not detected in the GP extract (refer to Table 2). Lower case letters indicate significant differences between cultivar-loaded masses (*Carménère* 1/*Merlot* 1/*Cabernet Sauvignon* 1; *Carménère* 2/*Merlot* 2/*Cabernet Sauvignon* 2, *Carménère* 3/*Merlot* 3/*Cabernet Sauvignon* 3).

## Data Availability

The data presented in this study are available in the article and supplementary materials.

## Supplementary Materials

The following supporting information can be downloaded at: https://www.mdpi.com/article/10.3390/antiox11102017/s1, Figure S1: Adsorption of anthocyanins (520 nm) and flavonoids (320 nm) compounds from *Carménère* (a,b), *Cabernet Sauvignon* (c,d), and *Merlot* (e,f) GP extracts on PVPP. Figure S2: Zone of inhibition generated by pomace extracts for *B. cereus* (a) and *P. syringae* pv. *actinidiae* (b). Table S1: Antibacterial activity (inhibition zone) of grape pomace extracts (at different masses) and phenolic compounds retained on PVPP.
