# Supplementary material for: Antioxidant Compound Adsorption in Polyvinylpolypyrrolidone from Chilean Carménère, Cabernet Sauvignon, and Merlot Grape Pomaces as Potential By-Products
Source: Antioxidants (Basel). 2022 Oct 12;11(10):2017. doi: 10.3390/antiox11102017 (PMC9598612; doi:10.3390/antiox11102017)
Supplement: Supplementary file 1 [file antioxidants-11-02017-s001.zip › antioxidants-1899514-supplementary.pdf]

Supplementary Material

Article

Antioxidant compounds adsorption in polyvinylpolypyrrolidone from Chilean *Carménère, Cabernet Sauvignon,* and *Merlot* grape pomaces as potential by-products.

Nelson Díaz [1], Pedro M. Aqueveque [1], Alejandro Vallejos-Almirall [2], Rudi Radrigán [3], María C. Zúñiga-López [4] and Christian Folch-Cano [1,*]

[1] Departamento de Agroindustrias, Facultad de Ingeniería Agrícola, Universidad de Concepción, Av. Vicente Méndez 595, Chillán , 3812120, Chile

[2] Grupo Interdisciplinario de Biotecnología Marina (GIBMAR), Centro de Biotecnología, Universidad de Concepción, Edmundo Larenas 64, Concepción, 4070386, Chile

[3] Centro de Desarrollo Tecnológico Agroindustrial (CDTA), Facultad de Ingeniería Agrícola, Universidad de Concepción, Av. Vicente Méndez 595, Chillán, 3812120, Chile

[4] Departamento de Química Inorgánica y Analítica, Facultad de Ciencias Químicas y Farmacéuticas, Universidad de Chile, Sergio Livingstone 1007, Santiago, 8380492, Chile

* Correspondence: chfolch@udec.cl; Tel.: +56-42-2207578

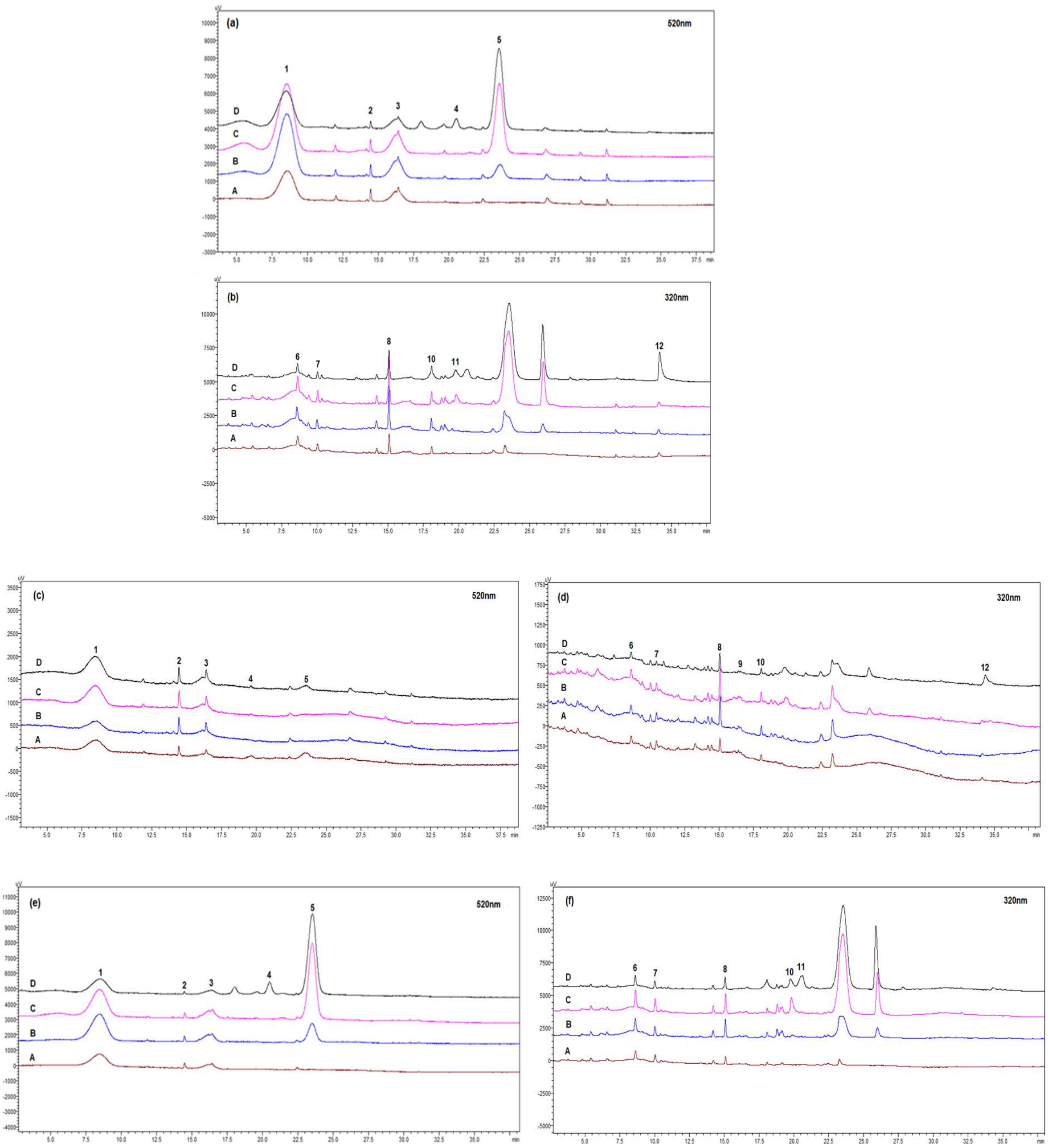

Figure S1. Adsorption of anthocyanins (520nm) and flavonoids (320nm) compounds from *Carmenere* (a,b), *Cabernet Sauvignon* (c,d), and *Merlot* (e,f) GP extracts on PVPP. Chromatogram "A" corresponds to the eluted extract from the adsorption process 1 to 4; "B" to the eluted extract from the adsorption process 5 to 8, "C" to the eluted extract from the adsorption process 9 to 12, and D to the GP extract. Peak numbers are: 1) Malvidin-3-O-glucoside + Peonidin 3-O-glucoside, 2) Delfinidin-3-O-caffeoyl-glucoside, 3) Malvidin-3-O-acetyl-glucoside, 4) Malvidin-3-O-caffeoyl-glucoside, 5) Malvidin-3-O-coumaroyl-glucoside, 6) Catechin, 7) Epicatechin, 8) Myricetin-3-galactoside, 9) Myricetin-3-O-glucoronide, 10) Quercetin-3-O-glucoside, 11) Myricetin-3-glucoside and 12) Quercetin.

Table S1. Antibacterial activity (inhibition zone) of grape pomace extracts (at different mass) and of phenolic compounds retained on PVPP.

	**Inhibición zone (mm)**															
**Microorganism**	**Carmenere (*µ*g)**			**Merlot (*µ*g)**			**Cabernet (*µ*g)**			**Quercetina (*µ*g)**			**PVPP+Ext (*µ*g)**	**Streptomycin (*µ*g)**		
**Gram-positive**	50	100	200	50	100	200	50	100	200	50	100	200	200	50	100	200
B. cereus	-	-	8.3±1.2	-	-	7.0±0.0	-	-	-	-	-	-	-	25.0±1.1	27.0±1.0	25±1.2
B. subtilis	-	-	-	-	-	-	-	-	-	-	-	-	-	-	-	-
S. aureus	-	-	-	-	-	-	-	-	-	-	7.0±0.0	7.0±0.0	-	20±1.1	22±1.1	22±1.2
L. monocytogenes	-	-	-	-	-	-	-	-	-	-	-	-	-	24±1.5	25±1.2	27±1.5
**Gram-negative**																
P. syringae pv. actinidiae	7.0±0.0	8.0±1.7	9.7±0.6	-	-	-	8.0±1.7	7.7±1.2	-	11.0±0.0	8.0±0.0	-	-	22±1.7	22±1.7	25±1.7
E. coli	-	-	-	-	-	-	-	-	-	-	-	-	-	27.0±0.0	27.0±0.0	27.0±0.0
Salmonella **sp.**	-	-	-	-	-	-	-	-	-	-	-	-	-	20.0±1.2	22.0±2.5	25.0±2.2

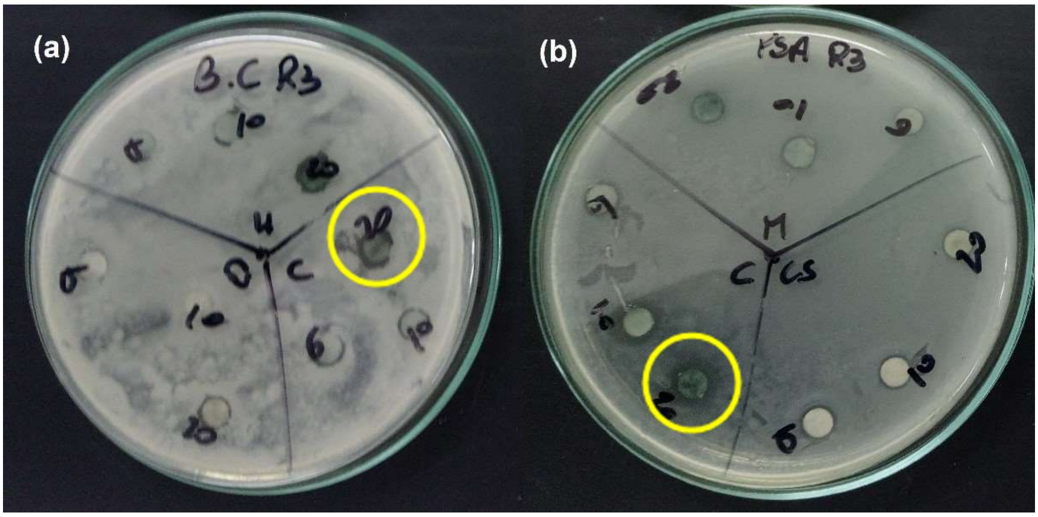

Figure S2. Growth inhibition generated by GP extracts on *B. cereus* (a) and *P. syringae pv. actinidiae* (b).
